# Usability and Feasibility of a Smartphone App to Assess Human Behavioral Factors Associated with Tick Exposure (The Tick App): Quantitative and Qualitative Study

**DOI:** 10.2196/14769

**Published:** 2019-10-24

**Authors:** Maria P Fernandez, Gebbiena M. Bron, Pallavi A Kache, Scott R Larson, Adam Maus, David Gustafson Jr, Jean I Tsao, Lyric C Bartholomay, Susan M Paskewitz, Maria A Diuk-Wasser

**Affiliations:** 1 Earth Institute Columbia University New York City, NY United States; 2 Department of Ecology, Evolution and Environmental Biology Columbia University New York, NY United States; 3 Department of Entomology University of Wisconsin-Madison Madison, WI United States; 4 Center for Health Enhancement System Studies University of Wisconsin-Madison Madison, WI United States; 5 Department of Fisheries and Wildlife Michigan State University East Lansing, MI United States; 6 Department of Pathobiological Sciences University of Wisconsin-Madison Madison, WI United States

**Keywords:** Lyme disease, ticks, ecological momentary assessment, citizen science

## Abstract

**Background:**

Mobile health (mHealth) technology takes advantage of smartphone features to turn them into research tools, with the potential to reach a larger section of the population in a cost-effective manner, compared with traditional epidemiological methods. Although mHealth apps have been widely implemented in chronic diseases and psychology, their potential use in the research of vector-borne diseases has not yet been fully exploited.

**Objective:**

This study aimed to assess the usability and feasibility of The Tick App, the first tick research–focused app in the United States.

**Methods:**

The Tick App was designed as a survey tool to collect data on human behaviors and movements associated with tick exposure while engaging users in tick identification and reporting. It consists of an enrollment survey to identify general risk factors, daily surveys to collect data on human activities and tick encounters (Tick Diaries), a survey to enter the details of tick encounters coupled with tick identification services provided by the research team (Report a Tick), and educational material. Using quantitative and qualitative methods, we evaluated the enrollment strategy (passive vs active), the user profile, location, longitudinal use of its features, and users’ feedback.

**Results:**

Between May and September 2018, 1468 adult users enrolled in the app. The Tick App users were equally represented across genders and evenly distributed across age groups. Most users owned a pet (65.94%, 962/1459; *P*<.001), did frequent outdoor activities (recreational or peridomestic; 75.24%, 1094/1454; *P*<.001 and 64.58%, 941/1457; *P*<.001, respectively), and lived in the Midwest (56.55%, 824/1457) and Northeast (33.0%, 481/1457) regions in the United States, more specifically in Wisconsin, southern New York, and New Jersey. Users lived more frequently in high-incidence counties for Lyme disease (incidence rate ratio [IRR] 3.5, 95% CI 1.8-7.2; *P*<.001) and in counties with cases recently increasing (IRR 1.8, 95% CI 1.1-3.2; *P*=.03). Recurring users (49.25%, 723/1468) had a similar demographic profile to all users but participated in outdoor activities more frequently (80.5%, 575/714; *P*<.01). The number of Tick Diaries submitted per user (median 2, interquartile range [IQR] 1-11) was higher for older age groups (aged >55 years; IRR 3.4, 95% CI 1.5-7.6; *P*<.001) and lower in the Northeast (IRR[NE] 0.4, 95% CI 0.3-0.7; *P*<.001), whereas the number of tick reports (median 1, IQR 1-2) increased with the frequency of outdoor activities (IRR 1.5, 95% CI 1.3-1.8; *P*<.001).

**Conclusions:**

This assessment allowed us to identify what fraction of the population used The Tick App and how it was used during a pilot phase. This information will be used to improve future iterations of The Tick App and tailor potential tick prevention interventions to the users’ characteristics.

## Introduction

### The Use of Mobile Health in Vector-Borne Diseases

In the United States, the number of adults who own a smartphone has been steadily increasing over time for every age group within the adult population. It is estimated that 81% of the adult population owns a smartphone compared with 15% of the population owning a normal mobile phone [1]. The ubiquity of smartphones provides a unique opportunity to gather and share information about health and disease, given the capabilities such as location sensing and software apps. As a result, mobile health (mHealth) technology is becoming an important part of health care service provision and is changing the way in which people use health information and communicate with health organizations and health professionals [2,3]. mHealth allows the general public to access a health service where and when they need it. In the context of public health, mHealth is particularly suited for patient education, disease self-management, and remote monitoring of patients [4]. Moreover, the use of mHealth can leverage smartphone features to turn them into research tools with the potential to reach a larger section of the population in a more cost-effective manner than traditional epidemiological methods and transform survey instruments into high-frequency (fine temporal resolution), spatially resolved data collection tools [5]. The widespread use of smartphone apps can be thought of as a 2-way communication channel between affected users and researchers. Although apps have been widely implemented in chronic diseases and psychology [4], their potential use in the research of vector-borne diseases has not yet been fully exploited. Most mHealth apps applied to vector-borne disease research have targeted mosquito-borne diseases [6-9] and only a few have targeted tick vectors. These are limited to the country level in Europe [10,11].

### Lyme Disease Risk

Lyme disease is the most commonly reported vector-borne disease in the United States, with 300,000 cases estimated per year [12,13], the majority of which are reported from the northeastern and north-central states [12]. In these areas, Lyme disease risk is determined by human exposure to infected *Ixodes scapularis* ticks, which can occur either peridomestically or within natural areas [13-18]. The seasonality of human cases mirrors that of *I. scapularis* nymphal activity; nymphs are abundantly active from May to early August, peaking in early-mid summer [12]. The association between human cases and nymphal activity can be in part attributed to the small size of nymphal ticks compared with adults, resulting in prolonged or undetected attachment. Human exposure to ticks depends on the density of infected ticks, but this association is strongly modified by local conditions, including human behavior [19,20]. Human behaviors that have been shown to influence tick exposure include the frequency and type of outdoor activities and adaptive responses following interactions with tick habitat [13,21,22]. In turn, prior exposure to ticks may trigger multiple behavioral responses to reduce exposure, such as avoidance of tick habitat and the use of personal protection measures [16].

The links between human activity, mobility patterns, and tick exposure, however, have not been well documented in Lyme disease–endemic areas of the United States, in part, because of methodological limitations. Traditional mechanisms of data collection of human behavior (eg, retrospective questionnaires) are subject to uncertainties in the degree to which findings can be generalized beyond the investigation depending on how and where they are administered [23]; surveys administered through an app can help expand the population surveyed at a lower cost. In addition, survey results are often affected by recall bias and do not capture specific intrasubject variability, particularly when participants are required to generalize an experience or behavior [23]. To address these issues, we developed a smartphone app, The Tick App [24], to (1) serve as a research tool to better understand human behaviors affecting tick exposure and (2) engage the general public in active tick prevention and reporting in different regions of the United States. As a research tool, it includes epidemiological surveys using *ecological momentary assessments* (EMA) to gather quantitative behavioral data within the context of a participant’s daily routine [25]. There is an increasing body of literature describing the use of smartphone app technology as a platform for EMA implementation, particularly within psychology and substance abuse research [23,26,27]. To our knowledge, however, this method has not been leveraged in assessing behavioral risk factors for Lyme disease.

### Objective

In this study, we report on the development and implementation of The Tick App in the United States. Our overall aim was to assess its usability and feasibility and identify what fraction of the population engaged in The Tick App to better interpret the external validity of the app-derived data. Information from this study will be used to improve future iterations of The Tick App and tailor potential tick prevention interventions to the user population.

## Methods

### Design of The Tick App (Phases 1 and 2)

The Tick App was developed from a preprototype named *GeoQuestion*, which was designed and evaluated during the spring and summer of 2017 (phase 1: preprototype [28]; Table 1). On the basis of this experience, we developed The Tick App (phase 2: prototype [28]; Multimedia Appendix 1). To align the design of the app with potential users’ characteristics, we framed the activities based on a roadmap for creating mHealth apps [29] (Table 1). Coding of The Tick App was led by the Center for Health Enhancement System Studies at the University of Wisconsin-Madison, and a prototype was completed by April 2018. After testing the prototype extensively, the app was available for download in May 2018 (Multimedia Appendix 1). The app is available for Android and iOS operating systems and can be downloaded from smartphone app stores at no cost (at the time of this publication). This study complied with the Consolidated Standards of Reporting Trials (Multimedia Appendix 2).

**Table 1:** Phases, objectives, activities, and timeline in the design process of The Tick App based on the roadmap proposed in a study by van Velsen et al for creating mobile health apps and the guidelines for monitoring and evaluating mobile health interventions developed by the World Health Organization.

Phases of development [29]	Objective	Activities	Timeline	World Health Organization phase [28]
Contextual inquiry	Identification of end users and the context in which the app will be used	Identify research objectives; focus groups with the users of GeoQuestion preprototype app	September to November 2017	Phase 1: preprototype
Value specification	Identification of end users’ values and requirements from phase 1	Design the content of The Tick App	December 2017 to January 2018	Phase 2: prototype design
Design	Creation and testing of prototype	Code The Tick App; pilot testing of prototype; focus groups in target study areas	February to April 2018	Phase 2: prototype design
Operationalization	Launch and recruitment of the app	Passive and active recruitment of users; collect data generated by The Tick App users	May to August 2018	Phase 3a: pilot the prototype (usability)
Summative evaluation	Gather feedback from The Tick App users	Focus groups with The Tick App users	September 2018	Phase 3b: pilot the prototype

### Functionalities and Workflow of The Tick App

**Figure 1:**
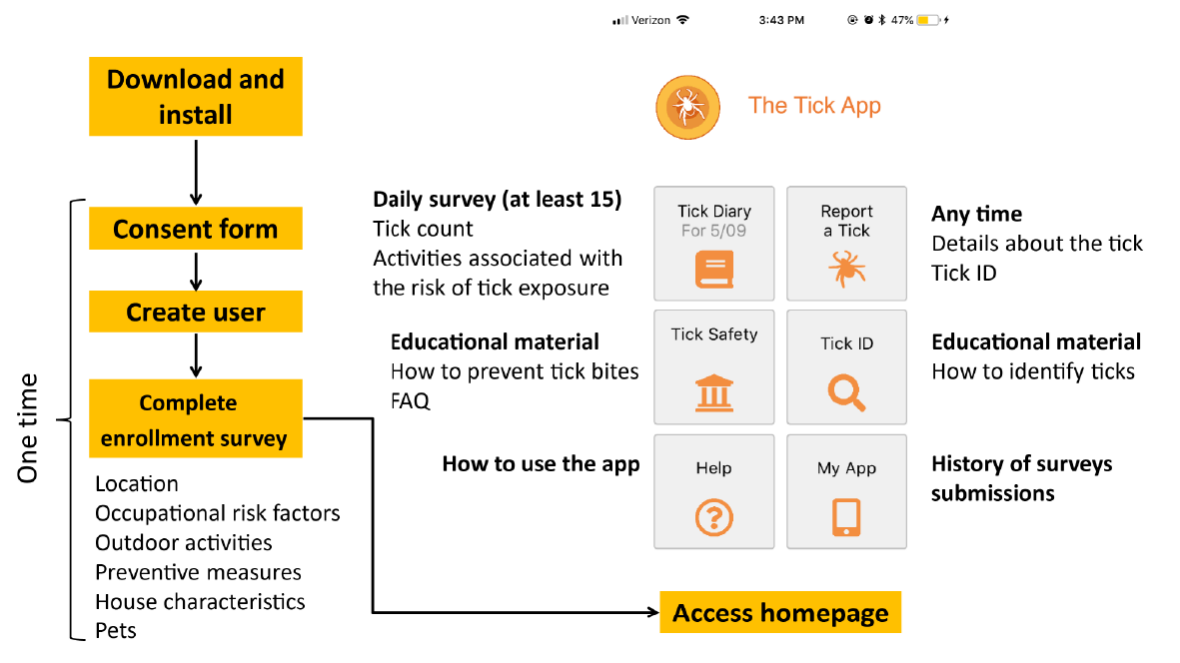
Workflow and homepage of The Tick App. The time frequency for the surveys (Tick Diary and Report a Tick) and the type of content of the remaining functionalities displayed on the homepage were indicated. FAQ: frequently asked question; ID: identification.

After users downloaded and installed The Tick App, a brief explanation of the study and electronic informed consent were displayed (Figure 1). The informed consent had to be accepted before proceeding to create a user account. The Tick App functionalities included an enrollment survey, which was modified from the preprototype. This survey was designed to take less than 10 min to fill out and aimed to collect the users’ demographic data, house characteristics, past experiences with ticks and tick-borne diseases, typical use of personal prevention methods and household interventions to reduce tick encounters, and general frequency of outdoor and peridomestic activities during the spring and summer. This survey was completed only once by the user before accessing the homepage.

On the homepage, several functionalities were available at any time of the day. In contrast, the *Tick Diary* was only available once a day (from 5 pm to 10 am CST; Figure 1). The *Tick Diary* was a daily retrospective survey that collected information on the user’s daily outdoor activities, tick encounters on themselves, their pets or members of their household, and any personal protection measures used to prevent tick bites. This survey collected fine-scale temporal data about human behavior in association with tick encounters, and it was designed to take less than 1 min to complete. We asked all users to submit at least 15 daily surveys to gain representation of the typical activity patterns for each individual, assess the variability between users, and calculate an accurate measure of the risk of tick encounter. A pop-up notification showed up every day at 5 pm CST until 15 *Tick Diaries* were completed. This study follows the recommended guidelines for reporting results of internet e-surveys (Multimedia Appendix 3).

Users were also able to report any ticks they found through the *Report a Tick* survey in the app and could send a picture of the tick via a Web-based form external to the app. These tick reports collected information on the tick encounter: who had the tick (ie, self, pet, or member of the household), if it was attached, and where the exposure might have occurred. Users were also asked to identify the tick from photographs that were provided in the app, including photos of female and male adult *Dermacentor variabilis*, *Amblyomma americanum,* and *I scapularis* as well as an *I scapularis* nymph. If users sent a picture of the tick via the online survey, trained members of our research group with experience on taxonomic identification of ticks identified the tick to the species and life stage visually from the picture and provided this information to the user via email. The confidence in the identification depended on the quality of the picture submitted and varied between stages (easier in adults compared with nymphs). On the basis of our experience, we were able to identify 94.8% (502/533) of pictures submitted to the species level with a high degree of confidence in the identification.

Through the app, we also provided educational resources about the biology and ecology of ticks (*FAQ* and within *Tick Safety*), how to identify them (*Tick ID*), and measures for protection against them (*Tick Safety*). All functionalities were accessible offline, including the surveys (*Tick Diary* and *Report a Tick*), which were stored locally on the smartphone, but needed an online connection to be submitted. Information about the number of surveys submitted, log out button, and the informed consent were available in *My App*. The *Help* functionality explained the use of the app to the users.

The Tick App was also able to use location services through the smartphone to capture the global positioning system (GPS) coordinates if the user allowed for this functionality. If location services were enabled, the app would record GPS coordinates every 15 min depending on the cell phone service. A more detailed explanation of the backend components of The Tick App and the surveys’ design can be found in the technical appendix (Multimedia Appendix 4).

### Usability of The Tick App (Phase 3a)

We evaluated the usability of The Tick App from April 20 to September 3, 2018 (phase 3a [28]; Table 1). We assessed our dissemination and recruitment strategies, the profile of The Tick App users with respect to their demographics, risk factors associated with tick exposure risk, and geographical distribution. We also evaluated the longitudinal use of The Tick App and how users engaged with different app functionalities.

#### User Enrollment

The Tick App was available to any adult aged older than 18 years who lived in the United States, owned a smartphone, and was familiar with software apps. Active and passive recruitment of users began with a special focus on the Midwest and Northeast, particularly in Wisconsin and southern New York state, respectively. At these locations, passive recruitment occurred by media coverage. Active recruitment was undertaken during house visits coupled with ongoing field research involving tick sampling in yards at selected study sites (Eau Claire, WI, and Staten Island, NY). During these visits, the researchers explained the objective of the app and invited residents to participate as users (Multimedia Appendix 5). Recruitment was conducted during June and July 2018. All activities complied with the ethical principles included in the Declaration of Helsinki as reviewed by Institutional Review Boards (IRBs) of Columbia University (IRB protocol: AAA3750-M00Y01) and the University of Wisconsin-Madison (IRB protocol: 2018-84). Participants provided their informed consent through the app. We assessed the longitudinal enrollment pattern during the study period and the effects of the active recruitment and media coverage (passive recruitment).

#### User Profile

To characterize the user profile, we considered demographic variables (age and gender), risk factors associated with tick encounters (type of property, owning a pet, and frequency of outdoor activities), and previous experience with ticks and tick-borne diseases. *Frequent peridomestic activity* described users who reported mowing the lawn or gardening once a week. *Frequent outdoor recreation* described users who went hiking, running, or biking on nature trails or visited the beach (at a lake or ocean) at least once a week or went hunting, fishing, bird watching, or camping at least once a month during the spring and summer months.

We used multiple correspondence analysis (MCA) as an exploratory analysis of the association among different user characteristics [30]. The MCA is the equivalent of principal component analysis but for categorical variables; it reduces the dimensionality of the covariance matrix into linear combinations of the original variables (dimensions) and decomposes the variance (inertia) of the sample [30]. The different dimensions can be assessed graphically using biplots, which allow a better understanding of how the variables are interrelated and their relative contribution to the score [31]. We used a first MCA including all users’ characteristics (age, gender, owning a pet, type of house, frequent outdoor recreation, outdoor work or volunteer job, and frequent peridomestic activity) to assess the association between the variables. We conducted a second MCA, including only owning a pet, frequent outdoor recreation, outdoor work or volunteer job, and frequent peridomestic activity, to construct a summary index of the frequency of outdoor activities (ie, the outdoor activity index). As the first dimension captures most of the inertia, the coordinate (value estimated for each individual based on their characteristics and which represents the contribution to the inertia in the study population) can be used as a quantitative index [32].

#### Geographic Distribution of Users

When assessing the geographic distribution of users in the United States, we considered the major census regions used by the US Census Bureau [33]. We evaluated if the user’s profile varied by region and the effects of the Lyme disease reporting status by county on the number of users. We used Lyme disease case data publicly available from the Centers for Disease Control and Prevention (CDC) [34] to estimate Lyme disease incidence at a county level. We used the number of cases reported (*confirmed* and *probable* cases) per county for a 5-year period (2013-2017) and the population size obtained from the National Census in 2010 to estimate a 5-year period Lyme disease annual incidence per county and percentage change in cases within that period. The 5-year period Lyme disease annual incidence was estimated as follows: (cumulative number of cases between 2013 and 2017)/(population size × 5 years) ×100,000. The mid-year total population was estimated as the population size in 2010 multiplied by the duration of the period [35]. We designated counties to be as *high incidence* if this measure was greater than 10 cases per 100,000, otherwise they were *low-incidence* counties or had *no cases of Lyme disease*. In addition, we used the CDC case data to classify the counties according to their percent change in Lyme disease incidence between 2013 and 2017. By combining this classification with Lyme disease incidence, we further classified the counties into 5 categories: (1) high incidence—no change; (2) high incidence—greater than 1-fold increase; (3) low incidence—no change; (4) low incidence—greater than 1-fold increase; and (5) no Lyme disease cases reported. A greater than 1-fold increase referred to counties where Lyme disease cases at least doubled during 2013 to 2017.

#### Longitudinal App Use

The data on the longitudinal use of the app were derived from the *Tick Diary*, *Report a Tick*, and screen views. Independent screen views (as opposed to continuous screen views) were considered as screen views at every 5-min intervals, assuming that screen views happening within 4 min or less represented a single interaction with The Tick App. We tested whether the user’s profile differed between recurring (ie, interacted with the app beyond the enrollment survey) and nonrecurring users and identified the most frequently used features of the app during the study period. Among recurring users, we evaluated the association between demographic variables, geographic location and outdoor activity pattern, and the number of *Tick Diaries* and tick reports submitted.

#### Statistical Analysis

To summarize the distribution of numeric variables (eg, number of users), we reported the interquartile range (IQR) as a measure of variability because numeric variables deviated from a normal distribution [36]. For descriptive bivariate analyses of categorical variables, we used chi-square tests and nonparametric Kruskal-Wallis tests when comparing categorical and continuous variables (eg, number of users vs region). We conducted multivariate analyses using generalized linear models [37] and generalized linear mixed models when accounting for random effects (eg, regional effects in the number of users per county) [38]. In the case of binary response variables, we used logistic regression models with logit as the link function and the relative risk expressed as odds ratios (ORs). When the response variable was numeric (count data), we used negative binomial models with log as the link function and the relative risk expressed as incidence rate ratios (IRRs). Negative binomial regression was preferred over Poisson regression, given the overdispersed distributions [39]; both models were compared by log-likelihood ratio tests. All analyses were implemented in Stata version 14.2 [40] and R version 3.2.3 (lme4 and car packages) [41].

### Feasibility Evaluation

In September 2018, we organized focus groups in Staten Island, NY, and Eau Claire, WI, by extending personal invitations to local users of The Tick App, with the goal of gathering feedback on the app after its implementation during the study period. In total, 14 users participated in the focus groups. The guiding questions used in the pre- and postimplementation focus groups can be found in Multimedia Appendix 6.

## Results

### User Enrollment

Between April 20 and September 3, 2018, 1468 (86.09%, 1468/1705) users completed the enrollment survey after downloading the app (Multimedia Appendix 7); 71.53% (1050/1468) users installed the app in smartphones operating with iOS, whereas 28.47% (418/1468) users installed it in smartphones operating with Android. User enrollment in the study mostly occurred from June to mid-July (1187/1468, 80.86% of the total number of users in the study period) and during the weekend (IRR 1.4, 95% CI 1.2-1.5; *P*<.001). Before the official app launch date (May 28, 2018), 81 users enrolled in the study, increasing to 209 within the first week of the official launch and to 521 by the end of the first week of active recruitment (Multimedia Appendix 7). Recruitment during household visits coupled with peridomestic tick sampling produced a 2-fold increase in enrollment on the same day of the visit and the following day, after accounting for weekend and media coverage effects (IRR 1.2, 95% CI 2.0-2.4; *P*<.001). Media coverage of the app also increased users’ enrollment by 2-fold on the same day and the day after the coverage (IRR 2.0, 95% CI 1.8-2.2; *P*<.001; Multimedia Appendix 7). The median time to complete the enrollment survey was 6.3 min (IQR 4.7-8.6).

### User Profile

The demographic profile of The Tick App users showed equal gender proportions and widespread age distribution, although young adults aged between 18 and 25 years were significantly underrepresented (Table 2). The median age was 48 years (IQR 36-60) and followed a bimodal distribution peaking at 37 years and 55 years (Multimedia Appendix 8). Users were significantly biased toward owning a pet (Table 2), and the majority of users with pets owned at least one dog (797/960, 83.02%). The proportion of users living in houses with yards was significantly higher than those living in apartments, and more than half of the users reported doing frequent peridomestic activities such as gardening and mowing the lawn during the spring and summer (Table 2). Regarding other outdoor activities, users doing at least one outdoor recreational activity once a week in urban parks or natural areas were significantly overrepresented (Table 2). In contrast, 644 out of 1454 users (44.29%) worked or volunteered in outdoor jobs (eg, landscapers and camp counselors), which was significantly less than the proportion who did not (Table 2).

**Figure 2:**
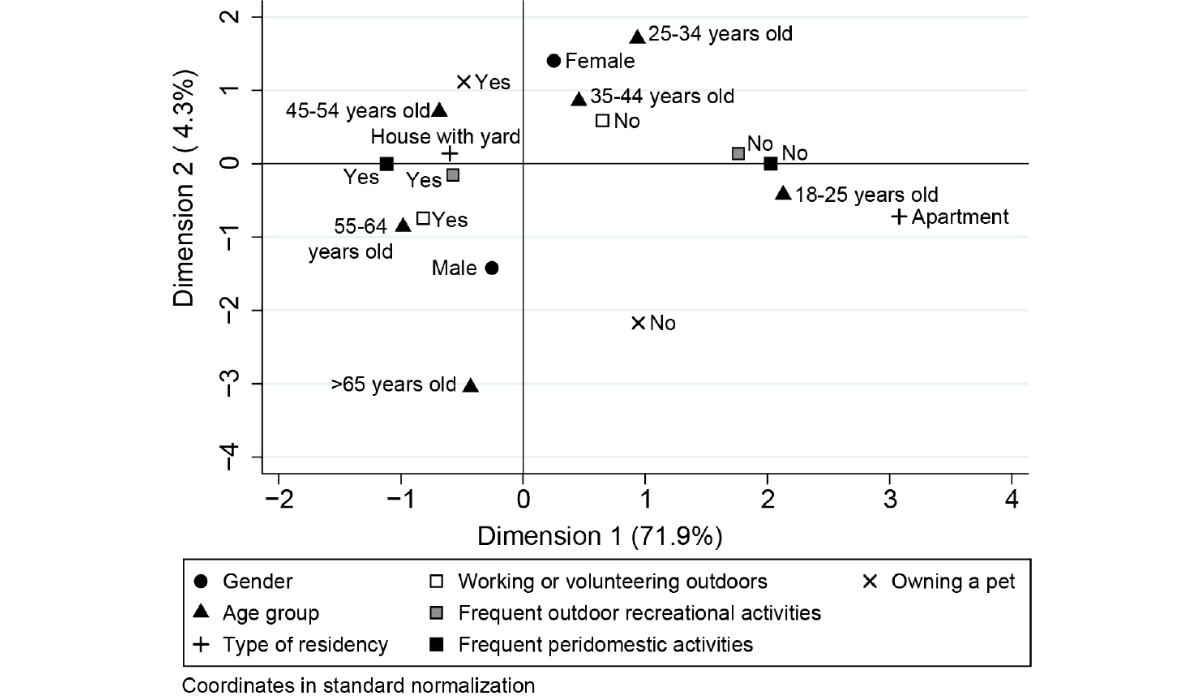
Biplot of the multiple correspondence analysis (MCA) of the users’ profile.

The exploratory MCA, which accounted for 76.2% of the total variability observed within the first 2 dimensions, showed that users reporting frequent outdoor recreational activities were also more frequently doing peridomestic activities at least once a week and working or volunteering outdoors (Figure 2, dimension 1), although the latter was slightly more associated with males than females (Figure 2, dimension 2). These outdoor enthusiasts were also more associated with owning a pet and with living in a house with a yard (Figure 2). Younger adults were associated with living in apartments and less often involved in outdoor activities in general, although they were underrepresented in the total number of users (Figure 2 and Table 2). Dimension 2 of the MCA separated users by their demographic characteristics (age and gender), but it only accounted for 4.3% of the variability, indicating that these variables did not contribute significantly to the differences observed among users. The outdoor activity index derived from the second MCA, which included only the variables that were associated in the first dimension of the previous MCA, accounted for 83.6% of the total variability (Multimedia Appendix 9). This index was significantly associated with a tick encounter in the previous winter or fall (logistic regression: OR 2.2, 95% CI 1.9-2.6; *P*<.001), but not with a previous diagnosis of a tick-borne disease (logistic regression: OR 1.16, 95% CI 0.98-1.38; *P*=.07) after adjusting for age and gender. One-third of users reported having a tick encounter the previous fall or winter when adult ticks are active, and half of the users reported finding a tick on their pet, whereas the percentage of users reporting a previous tick-borne diagnosis was comparatively lower (11.82%, 173/1464; Table 2). Nonetheless, the self-reported cases of Lyme disease in the previous year (2.05% of the users, 30/1461) were still considerably higher than the percentage of confirmed cases in 2017 within the total population from the counties where the majority of users live (median 0.06%, IQR 0.02%-0.10%).

**Table 2:** Users’ profile including demographic variables, type of property, frequent outdoor activities (occupational, recreational, and peridomestic), and previous experience with ticks and Lyme disease, as reported in the enrollment survey.

Variables		Users, n (%)	*P*^a^ value	Chi-square (*df*)
**Gender (n=1463)**			**.85**	**0.0 (1)**
	Male	720 (40.05)		
	Female	726 (49.46)		
	Others/prefer not to say	22 (1.50)		
**Age (years; n=1457)^b^**			**.001^c^**	**126.0 (5)**
	18-24	94 (6.43)		
	25-34	265 (18.13)		
	35-44	321 (22.96)		
	45-54	274 (18.74)		
	55-64	319 (21.82)		
	≥65	189 (12.93)		
**Pet owner** **(n=1454)**			**<.001^c^**	**146.8 (1)**
	Yes	962 (65.94)		
	No	497 (34.06)		
**Type of house** **(n=1455)**			**<.001^c^**	**0.0 (4)**
	House with yard	1109 (75.96)		
	Apartment	238 (16.30)		
	Cabin/cottage	65 (4.45)		
	Mobile home	22 (1.51)		
	Other	26 (1.78)		
**Work or volunteer outdoors** **(n=1454)**			**<.001^c^**	**18.9 (1)**
	Yes	646 (44.28)		
	No	813 (55.72)		
**Frequent outdoor recreation** **(n=1449)**			**<.001^c^**	**368.8 (1)**
	Yes	1094 (75.24)		
	No	360 (24.76)		
**Frequent peridomestic activities** **(n=1452)**			**<.001^c^**	**122.7 (1)**
	Yes	941 (64.58)		
	No	516 (35.42)		
**Tick exposure in the previous fall** **(n=1458)**			**<.001^c^**	**201.5 (1)**
	Yes	459 (31.37)		
	No	1004 (68.63)		
**Previous tick-borne disease diagnosis** **(n=1459)**			**<.001^c^**	**849.0 (1)**
	Yes	173 (11.82)		
	No	1291 (88.18)		
**Tick finding in their pet during the previous fall** **(N=958)**			**.004^d^**	**8.1 (1)**
	Yes	525 (54.57)		
	No	437 (45.43)		

^a^*P* values of the chi-square test of H_0_=equal distribution among users are presented.

^b^For age, we tested H_0_=equal distribution compared to the total US population (2016 population estimates).

^c^*P*<.001.

^d^.001≤*P*≤.05.

### Geographic Distribution of Users

**Figure 3:**
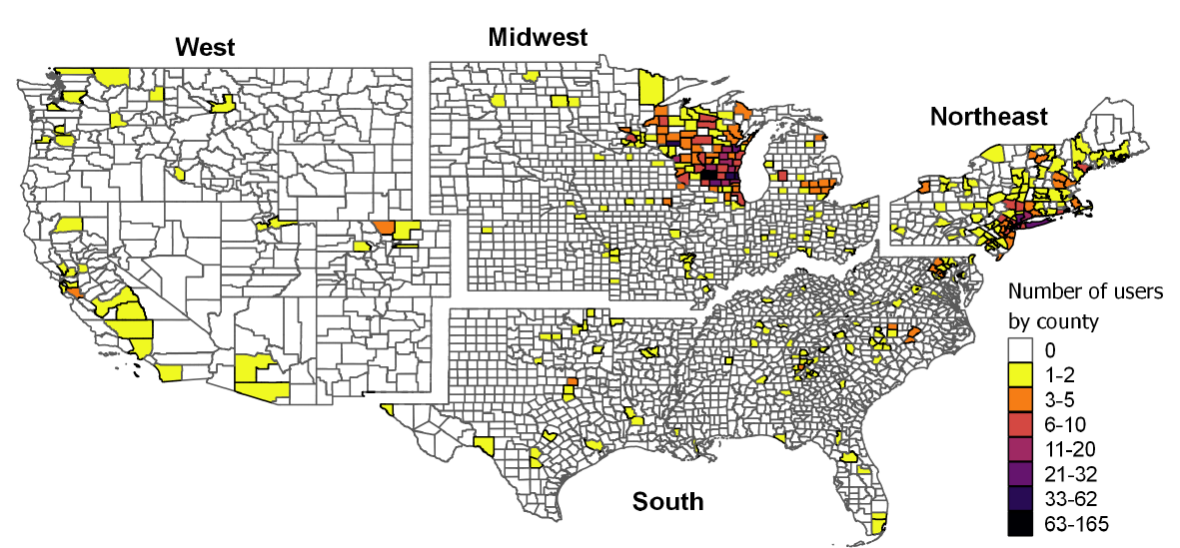
Number of users per county and per region for the United States by the major census regions in the United States.

**Figure 4:**
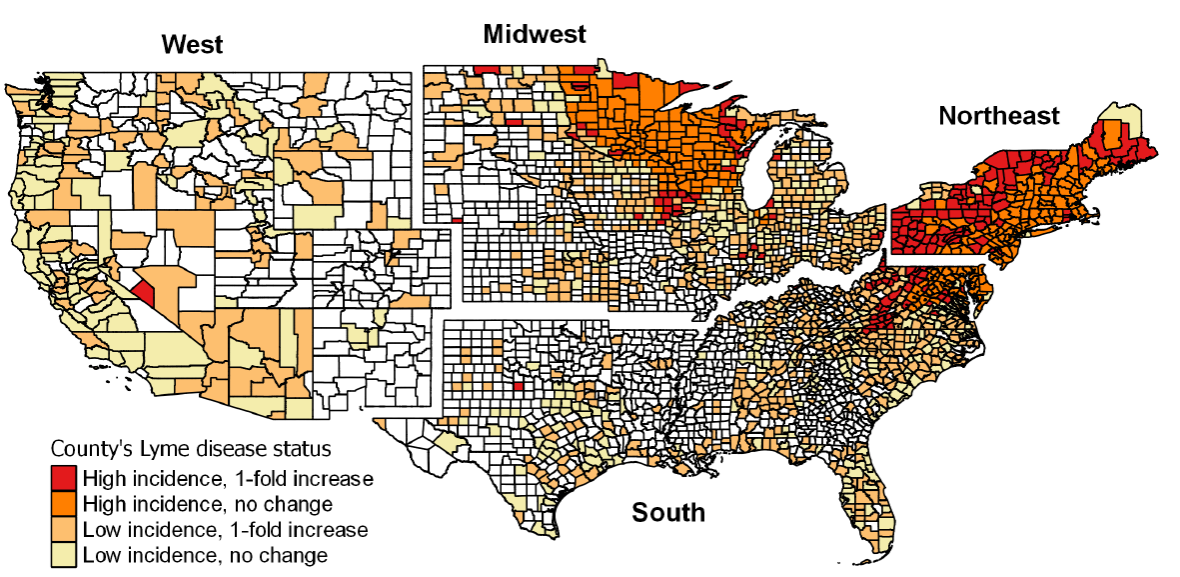
County’s Lyme disease status according to Lyme disease incidence and recent increase in 2013-2017.

**Figure 5:**
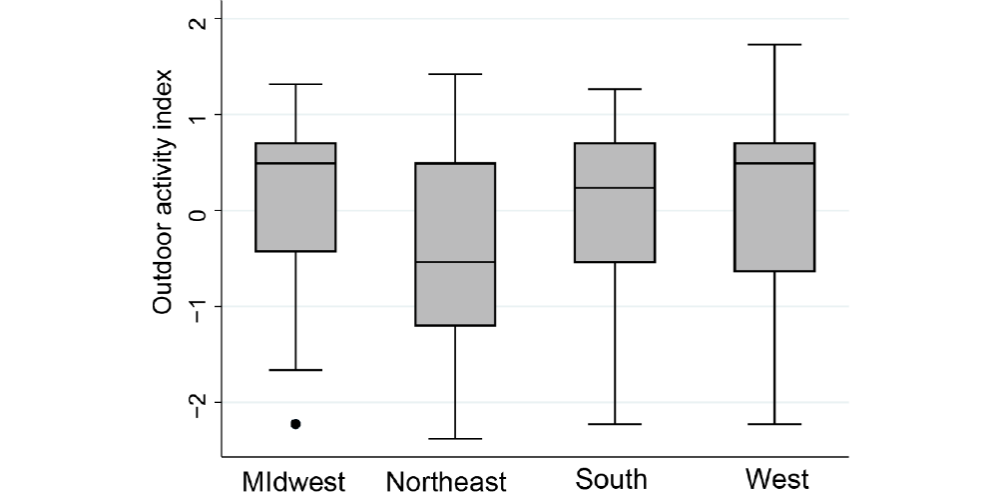
The outdoor activity index derived from the multiple correspondence analysis (MCA) excluding age and gender, by the major census regions in the United States.

We observed a nationwide distribution of users within the United States, although most of them lived in the Midwest (824/1457, 56.55%), followed by the Northeast (481/1457, 33.01%), and a smaller fraction of users lived in the South and West regions (107/1457, 7.34%, and 45/1457, 3.09%, respectively; Figure 3). Within the Midwest, 82.3% (682/824) of users lived in Wisconsin, whereas users from the Northeast mostly lived in southern New York and New Jersey (305/481, 63.4%, and 65/481, 13.5%, respectively), consistent with the area of influence of our study and recruitment efforts. Most of the users came from high-incidence counties with no recent changes in the number of Lyme disease cases (71.7%, 1045/1457), followed by low-incidence counties (24.9%, 363/1457) and counties with no reported cases (3.4%, 49/1457) (Figure 4). A higher proportion of users in the Northeast downloaded The Tick App on iOS and Android, compared with the Midwest (389/481, 80.9% and 534/824, 64.8%, respectively; χ^2^_1_=37.8; *P*<.001). Although the demographic profile was similar across regions, the outdoor activity index differed (Kruskal-Wallis test, χ^2^_3=_93.6; *P*<.001) and was lower in the Northeast compared with the other regions (Figure 5). Finally, the number of users per county was higher in those with a larger population size and high Lyme disease incidence or with low incidence but a 1-fold increase in the number of reported cases in the previous 5 years (2013-2017), after adjusting for regional random effects (Figure 4 and Table 3).

**Table 3:** Generalized linear mixed model for the number of users per county versus the county population size and the Lyme disease status of the county based on Lyme disease incidence and percent change of cases from 2013 to 2017, adjusting for the regions’ random effects. We used a negative binomial model, and the effect of each independent variable is expressed as incidence rate ratios.

Variables^a^		Incidence rate ratio	95% CI	*P* value
County population size (per 100,000)		1.3	1.2-1.4	<.001^b^
**County Lyme disease status (2013-2017)**				
	No Lyme disease cases reported	1	—^c^	—
	Low incidence—no change	0.8	0.4-1.7	.60
	Low incidence—greater than 1-fold higher increase	1.8	1.1-3.2	.03^d^
	High incidence—no change	4.2	2.1-8.1	<.001^b^
	High incidence—greater than 1-fold higher increase	3.5	1.8-7.2	<.001^b^

^a^Region (random effect) coefficient=0.6 (95% CI 0.1-4.9); Log-likelihood ratio test, *P*<.001.

^b^*P*<.001.

^c^Not applicable (reference category).

^d^.001≤*P*≤.05.

### Longitudinal App Use

**Figure 6:**
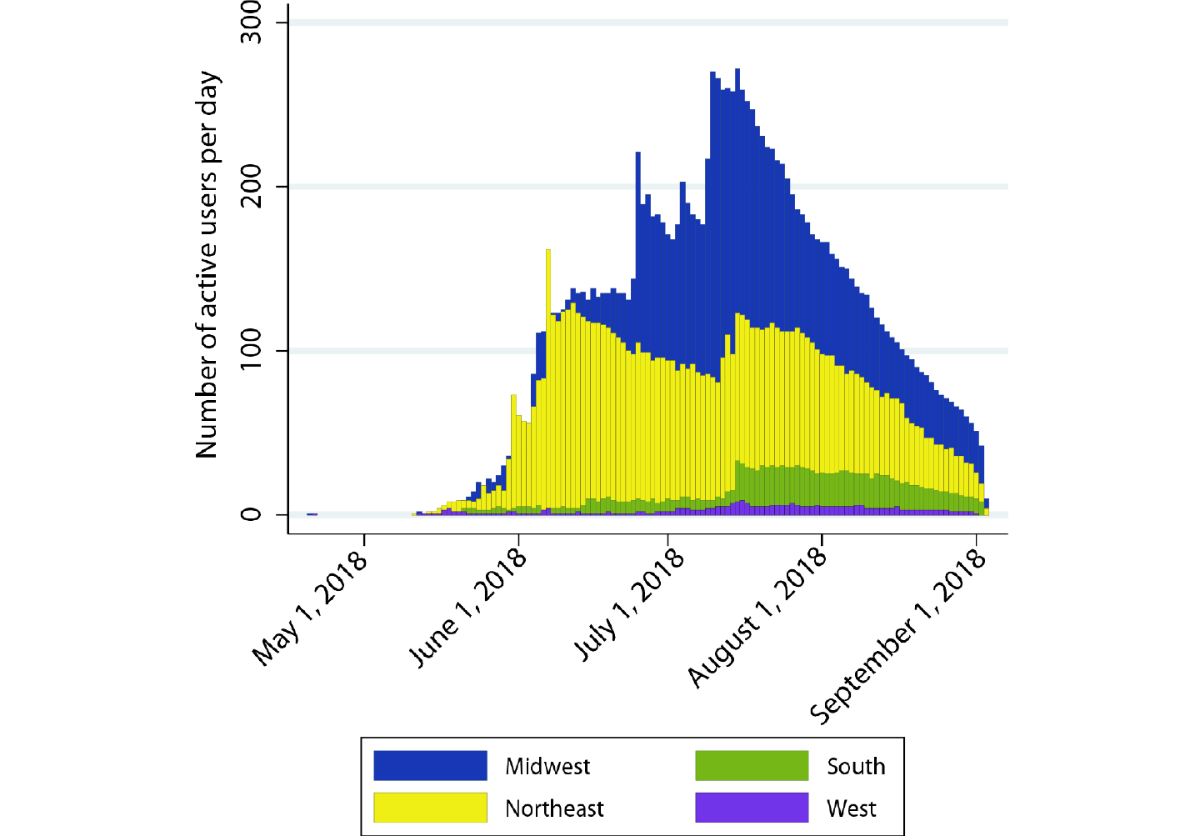
Number of active users per day between May and September 2018, by region.

After completing the enrollment survey, 49.25% (723/1468) of the users continued to interact with The Tick App. Users interacted with the app for a median of 25 days (IQR 12-49), 17.5% (126/723) interacted for up to 1 week, 19.0% (138/723) interacted between 1 week and 15 days, and 63.5% (459/723) interacted for 15 days or more. The median time that users interacted with the app per day was 1.9 min (IQR 0.7-4.7), and the median number of total users per day was 251 (IQR 109-291), peaking in June and mid-July and declining steadily thereafter in both the Midwest and Northeast (Figure 6), consistent with a decline in nymphal activity.

The demographic profile of recurring users compared with nonrecurring users did not vary with gender or age, but users were more likely to use the app after completing the baseline survey if they worked or volunteered outdoors and if they did outdoor recreational activities frequently, although no effect was observed if they did frequent peridomestic activities (Multimedia Appendix 10). A higher proportion of recurring users reported having had a previous tick bite compared with nonrecurring users (268/721, 37.2% vs n=191/742, 25.7%, χ^2^_1_=22.1; *P*<.001), but no differences were observed in the proportion of self-reported previous diagnosis of a tick-borne disease (95/722, 13.2% vs 78/742, 10.5%, χ^2^_1_=2.4; *P*=.12). Similarly, the number of follow-up days per user increased only with the outdoor activity index (IRR 1.2, 95% CI 1.1-1.4; *P*<.01), after adjusting for age, gender, and the region, indicating that users were more likely to use the app if they did outdoor activities frequently.

**Figure 7:**
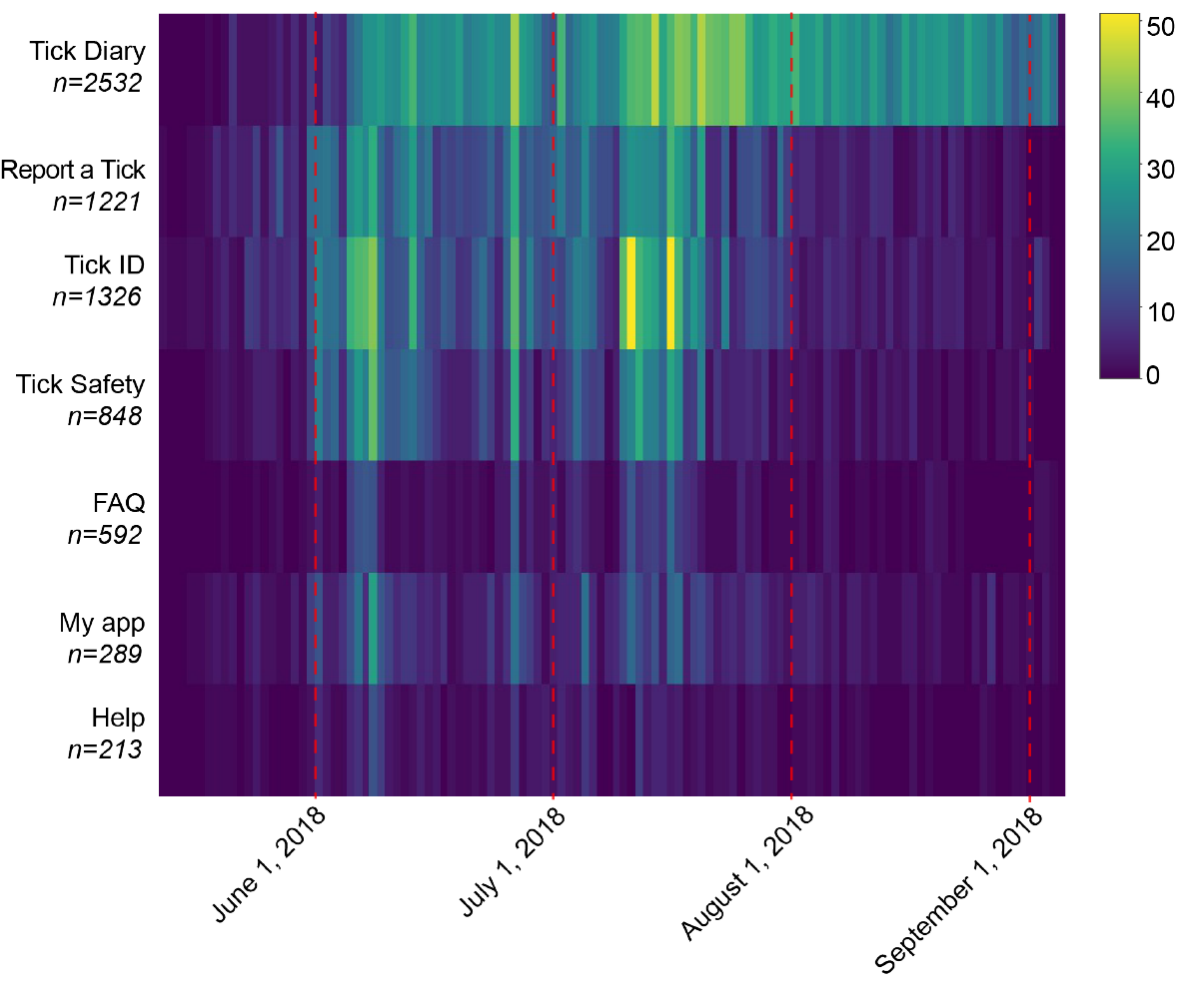
Heatmap of the independent screen views (every 5 min) per functionality, for all users between May and September 2018. For each day and functionality, the lighter color (see color scale) indicates a higher number of independent screen views. The dashed red lines indicate the first day of each month for reference.

On the basis of the unique screen views every 5 min (n=7021), the *Tick Diary* was the most frequently used feature in The Tick App, followed by *Report a Tick* and *Tick ID* (Figure 7). Educational material (*Tick safety*, including FAQ) was less popular among users, and app-related information (*Help* and *My App*) was the least used feature (Figure 7). The *Tick Diary* use was sustained during the study period, peaking in mid-July, following a peak in active users (Figures 6 and 7). *Report a Tick*, *Tick ID*, and *Tick Safety* had a more variable longitudinal use and were most frequently used in early June and mid-July (Figure 7).

At least one *Tick Diary* was completed by 50.8% (367/723) of the recurring users, and one-fourth of the total users enrolled in the app. The proportion of recurring users completing *Tick Diaries* for at least 1 week was 32.2% (118/367), whereas 21.8% (80/367) of recurring users completed at least 15. In total, 367 *Tick Diaries* were completed during the study period, with a median number of 2 submitted *Tick Diaries* per user (IQR 1-11). The median time to complete and submit each *Tick Diary* was 34 seconds (IQR 22-63). The users reported a tick encounter on themselves in 24.5% (90/367) of the *Tick Diaries* submitted, and a total of 149 tick encounters, which resulted in 12 tick encounters per 1000 person-days. In addition, tick encounters on pets were reported in 17.0% (46/270) of *Tick Diaries*, whereas tick encounters on a member of the family were reported in 13.4% (43/320) of *Tick Diaries*.

Similar to the *Tick Diary*, approximately half of the recurring users (381/723, 52.7%) and one-fourth (381/1468, 26.0%) of the total number of enrolled users submitted at least one *Report a Tick* survey. However, they were not always the same recurring users completing both surveys; only 42.5% (162/381) of the users who completed at least one *Tick Diary* also submitted a tick report using the *Report a Tick* functionality. In total, 650 tick reports were submitted, with a median number of 1 report per user (IQR 1-2). The median time to complete *Report a Tick* was 1.8 min (IQR 1.1-3.3). Although the number of tick reports per user increased significantly with the outdoor activity index and women submitted more *Tick Diaries* than men, the number of *Tick Diaries* completed did not vary significantly with the frequency of outdoor activities or gender (Table 4). The number of both *Tick Diaries* and tick reports was significantly higher in older age groups (Table 4). Regional differences in the number of *Tick Diaries* per user were observed; users living the Midwest submitted more *Tick Diaries* than completed users in the Northeast, although these regional differences were not observed in the number of tick reports (Table 4).

**Table 4:** Generalized linear model for the number of Tick Diaries and tick reports submitted per user versus demographic variables, location (region), and frequency of outdoor activities (outdoor activity index). We used a negative binomial model, and the effect of each independent variable is expressed as incidence rate ratios (N=712).

Variables		Number of *Tick Diaries*			Number of tick reports		
		IRR^a^	95% CI	*P* value	IRR^a^	95% CI	*P* value
**Gender**							
	Female	1	—^b^	—	1	—	—
	Male	0.9	0.6-1.3	0.66	0.7	0.5-0.9	.01^c^
**Age (years)**							
	18-25	1	—	—	1	—	—
	25-35	1.1	0.5-2.5	.86	1.8	0.9-3.6	.07
	35-45	1.2	0.5-2.7	.75	1.4	0.7-2.7	.32
	45-55	1.3	0.6-3.1	.49	1.3	0.6-3.1	.48
	55-65	3.4	1.5-7.6	<.001^d^	1.6	0.8-3.1	.15
	≥65	3.8	1.6-9.2	<.001^d^	2.3	1.1-4.5	.02^c^
**Region**							
	Midwest	1	—	—	1	—	—
	Northeast	0.4	0.3-0.7	<.001^d^	1	0.7-1.3	.98
	South	0.5	0.2-1.1	.10	1	0.6-1.6	.96
	West	0.3	0.1-1.3	.10	1.2	0.4-3.2	.73
Outdoor activity index		1.1	0.9-1.4	.41	1.5	1.3-1.8	<.001^d^

^a^IRR: incidence rate ratio.

^b^Not applicable (reference category).

^c^.001≤*P*≤.05.

^d^*P*<.001.

### Feasibility Evaluation

We identified 4 major themes from the feedback provided by users at the end of the study period: (1) effective communication, (2) content, (3) operability, and (4) incentives. Effective communication referred to our ability to effectively communicate the goals of the study and the intended use of the app. We found that some users were confused about completing the *Tick Diaries* and thought they only had to complete one when they found a tick, although we requested 15 consecutive *Tick Diaries* regardless of tick encounter. The main reasons why we failed to communicate effectively appeared to be the name of the functionality (ie, *Tick Diaries* included the word Tick and that was misleading) and the insufficient explanation before accessing the homepage of the app. Although this information was included in the Help functionality, it was rarely accessed by users. Users participating in the focus groups agreed that the educational content was complete, but some mentioned some difficulties in navigating the app to find some of the materials they were most interested in (eg, how to remove a tick). They mentioned greater interest in practical resources than in general knowledge about ticks and tick-borne diseases. Regarding operability, users mentioned that it was easy to navigate and access, although some issues about the content organization were mentioned. In general, the operability of The Tick App matched the skills of the users and did not require special training. Finally, as in the preoperationalization feedback, users mentioned that they would like to access more local data regarding the risk of tick encounters and mentioned mapping reported ticks in their neighborhood as a highly desirable functionality.

## Discussion

We used an iterative mixed-methods approach, in which findings from each step informed subsequent steps, to develop and evaluate the implementation of The Tick App, the first theory- and evidence-based smartphone app available in the United States to focus on ticks and tick-borne diseases. The Tick App users were equally represented across genders and evenly distributed across age groups; most users owned a pet, did frequent outdoor activities (recreational or peridomestic), and lived in the Midwest and Northeast regions in the United States, more specifically in Wisconsin, southern New York, and New Jersey. The number of users was higher in high-incidence counties for Lyme disease and in those counties with cases recently increasing. Half of the users were recurring users who participated in outdoor activities more frequently.

Within the wide range of citizen science projects, The Tick App can be classified as *contributory* because the public is mostly involved in data collection [42]. Owing to the public’s voluntary role, it is imperative to appeal to the users’ needs and evaluate the uptake and longitudinal use of the app to validate its use as a research app. In this study, qualitative analyses were used to test and adapt multiple versions of the app to maximize users’ experience. In line with van Velsen et al [29] who identified the requirements for a smartphone app to engage with the public in the prevention of tick bites in the Netherlands, The Tick App included a functionality to allow users to report ticks, videos with information on how to remove an attached tick, and alerts to remind people to check for ticks at the end of the day. The latter was also coupled with an alert to complete the daily activity survey, in line with the EMA method used in other human behavioral studies [43]. Quantitative analyses of the data actively submitted by the users and passively collected by the app (ie, usage data) were used to assess potential biases in the data collected and evaluate the factors associated with the uptake and longitudinal use of the app.

The Tick App is unique in its design to provide a bidirectional flow of tick-related information (from the user to the researcher and vice versa). Users were able to receive tick identification services and educational information. In turn, our research team was able to combine tick reports with epidemiological data related to human behavior to better understand the risk of tick exposure. Other tick-related smartphone apps have been implemented in Europe, either by research teams (Tekenbeet and Tekenscanner in the Netherlands) [10,11] or public health agencies (Zecke in Switzerland, Signalement TIQUE in France, and TekenNet/TiquesNet in Belgium) [44-46]. These apps mainly focus on educational materials and tick surveillance from user reports but do not describe specific research objectives related to the behavioral risk factors of tick exposure. In addition, these apps are intended to be used at the country level [29]. In the United States, commercial apps focus on automated tick identification (What’s My Tick and Detectick) [47,48] and mapping of tick reports by users (TickTracker) [49]. The latter creates heatmaps (ie, density maps) of tick reports but without a validation procedure to ensure the reliability of the data, that is, the species identification and whether the specimen is a tick. With the exception of Tekenbeet [10,29], no publicly available information is provided for the other apps, which describes the methods and considerations taken in the design of the app or in the uptake and usage by the general public.

The geographic distribution of The Tick App users indicated that we were targeting the population residing in regions with the greatest risk of tick exposure to *I scapularis* ticks. Although The Tick App was available nationwide, the enrollment of users was higher in the Northeast and Upper Midwest regions in the United States, where the majority of the Lyme disease cases were reported [12]. Within these regions, uptake of the app was highest in those counties with high Lyme disease incidence and with recent increases in the number of reported cases. Nonetheless, the uptake of the app further coincided with those areas where we conducted active and passive recruitment of participants (Wisconsin and New York State), indicating that the population exposed to a higher risk of tick encounters is not necessarily actively searching for a smartphone app to help them manage the risk but is receptive to its use. We also observed a higher proportion of iOS users compared with Android users, although the market share trend was the opposite as of 2019 [50], which could indicate differences in socioeconomic status, age, and use pattern [51].

According to the COM-B system, a human behavioral model that emerged from a systematic review of behavior change theories [52,53], enrollment in the app may depend on the opportunities provided by the app (functionalities that appeal to the users’ needs), capability (confidence in using the app or check for ticks), and motivation (risk perception, sense of empowerment, or expectation of reward). When analyzing the user profile, the majority of users did report frequent outdoor recreational and peridomestic activities, and half of the users also reported outdoors occupations, which have previously been associated with the risk of tick encounters [13,14,16,22]. In this study, because of the high correlation between the variables related to outdoor exposure, we created an overall index of frequent outdoor activities, which was also found to be associated with self-reported tick encounters. Self-reported previous tick exposure of the app users was slightly higher (31.4%) than those reported in the national HealthStyles surveys for the Upper Midwest and Northeast (22.6%-29.8%), in which respondents were randomly recruited from a large, nationally representative panel of adults aged 18 years or older [20]. However, when comparing self-reported diagnosis with previous tick-borne disease diagnosis, the proportion of users in the app (11.8%) almost doubled that of respondents in the HealthStyles surveys (2.0%-6.5%) [20], and the proportion of users reporting Lyme disease cases in the previous year was higher compared with the general population in the counties where the users lived. These results indicate that The Tick App users are biased toward those with a previous self-reported diagnosis with a tick-borne disease rather than toward those with previous self-reported tick exposure. However, self-reported Lyme disease diagnosis might be higher than reported and estimated cases [54], and previous tick encounters will depend on the user’s frequency of checking for ticks after being outdoors and their ability to detect and identify a tick. Nonetheless, we achieved a broader representation of the general population compared with the Tekenbeet app in the Netherlands, where they found that 90.9% of their users reported having had a previous tick encounter and 56.4% reported previous Lyme disease diagnosis. In The Tick App, we observed no other strong demographic bias when analyzing the users’ profile, whereas Tekenbeet was slightly more biased toward females (56.8%).

Analysis of the longitudinal use of The Tick App indicated that its use was seasonal and coincided with the trend observed for google searches for *Lyme disease* and *tick bites* [55]. In the case of The Tick App, 49.2% of initial users continued using the app beyond enrollment compared with 67.5% of Tekenbeet users in the Netherlands, which has very similar features (tick reports and educational material) but does not include any surveys to collect behavioral information or other risk factors. Continued use of The Tick App was also related to the frequency of outdoor activities. Motivation to enroll and initial participation in citizen science projects have been found to be related to motivations pertaining to one’s own welfare (*egoism*), whereas increasing welfare of others and of the group seemed to play an important role (*altruism* and *collectivism*, respectively) for continuing participation [42]. *Altruism* and *collectivism* seemed to be important motivational drivers, given that the *Tick Diary* was the most frequently used functionality and it does not directly reward the user; however, the mean number of *Tick Diaries* submitted per person did not achieve our research goals (15 complete *Tick Diaries* per user). From the users’ feedback at the end of the study period, we identified 2 main barriers: (1) the objective was not clear to participants and (2) we needed new motivational strategies to sustain engagement of the initial participants. The *egoism* motivational category for continued use of The Tick App was still of considerable importance: the *Report a Tick* and *Tick ID* features were among the most frequently used as well. Although the former feature is also intended for our research goals, the associated online picture submission and tick identification services provided by the research group could be the primary motivation for the user. The user profile differed between those engaged with the research via *Tick Diaries* and those engaging via *Report a Tick*. Users completing *Tick Diaries* were older (aged 55 years and older) and might have more time to participate, whereas users completing tick reports engaged more often in outdoor activities. The challenge is to respond to the different motivational factors at different points of participation to keep users engaged and achieve a broader representation of the population at risk of tick bites.

Additional incentives for The Tick App could help increase enrollment and engagement but need to be carefully selected because they can introduce potentially unintended consequences [56]. One functionality that users mentioned that they would like in the app was a map showing the ticks that have been reported in their area. The TickTracker app allows users to map the ticks that have been reported through the app but lacks scientific quality control of the reports. There are 2 main issues with mapping self-reported data if the users are going to rely on this information for decision making: (1) the number of ticks reported will depend on the number of users in the area and (2) validation of tick reports (ie, confirming that specimens are ticks and determining which species is being reported) currently requires considerable time and human resources. Quality maps of tick distribution at a small scale (ie, county) would require a large number of validated tick reports provided by a large number of users continuously using the app. Mapping incomplete data from areas with few users and few reports could result in a false sense of safety. On the other hand, mapping unvalidated reports could result in a false sense of risk and create unnecessary anxiety in the user. An alternative to near real-time mapping of tick reports is providing an indicator of tick activity based on the location and seasonality, a strategy that was implemented in the Tekenbeet app at a national level. We are planning on incorporating this feature at a county level in a future iteration of The Tick App (The Tick App 2.0).

Tick identification seemed to be one of the main incentives for using the app, although picture submission was done externally to the app. In The Tick App 2.0, users will be able to submit a photo of the tick within the app to reduce the effort from the user endpoint. Automated tick identification built into the app would greatly reduce the resources invested in tick identification. However, the apps offering this functionality are either unclear about the validation procedures of the classification algorithm and certainty of tick species identification from a photo (What’s My Tick) or do not offer tick identification to the species level (Detectick). The incentives to complete the *Tick Diaries* (the research aspect of the app) may require other types of incentives [43]. Gamification or the use of game design elements (badges, leaderboards, rewards, and avatars) can help maintain user engagement by “harnessing the desire for competition and the goal-driven aspects of human nature” [56,57]. In The Tick App 2.0, we will incorporate badges that can be earned as daily surveys are completed and a progress bar so that the users can track their progress toward completing a 7-day streak, a 15-day streak, and a 21-day streak. These streaks were established based on the results from the analysis of the longitudinal usage: approximately 30% of returning users completed at least 7 *Tick Diaries* and approximately 20% completed at least 15 *Tick Diaries*. The minimum of 7 days will allow for each day of the week to be represented and allow us to study daily fluctuations in activity [43]. We also changed the name to *Daily Log* because *Tick Diary* was understood by some users as only meant to be completed when a tick was found, and a clearer explanation of the objectives was also included in the app. Finally, monetary incentives can also be considered, particularly to increase representation among less motivated groups [23,56]. To achieve a broader representation of the daily activities and the risk of tick encounters in the general population, monetary incentives could be useful at a manageable scale and for a specific period and would increase the external validity of our results. A downside of monetary incentives is that they might result in data fabrication for monetary gain [56]; however, Bell et al [58] found that offering incentives to complete regular surveys through an app did not encourage false responses when the same reward was offered regardless of the answer, although 1 response required less information (and thus, less effort from the user).

A common goal for many mHealth studies is the “identification of behaviors associated with health outcomes so that behavior change interventions may be designed and implemented on a large scale” [59]. The analysis of the enrollment and use of The Tick App helped us identify the successful aspects of the app as well as its limitations and potential biases that could limit the extrapolation of the results derived from the data collected. The Tick App also offers the opportunity to explore interventions oriented to reduce the risk of tick-borne diseases by increasing self-awareness and encouraging the use of protective measures [10,23,60]. Understanding who, how, and when people are using The Tick App would help us tailor the content of an intervention to achieve a greater effect. Finally, there is a need to continually evaluate and revise the app based on what users are willing to do and what they can expect in return, while meeting the data requirements for the research on the behavioral risk factors of human-tick encounters.

## Appendix

Multimedia Appendix 1 Phase 1 and 2: Pre-prototype and prototype design.

Multimedia Appendix 2 Consolidated Standards of Reporting Trials electronic health checklist.

Multimedia Appendix 3 Checklist for Reporting Results of Internet E-Surveys.

Multimedia Appendix 4 Technical appendix of the backend components of The Tick App.

Multimedia Appendix 5 Informed consent and recruitment material.

Multimedia Appendix 6 Guiding questions for focus groups conducted prior and after The Tick App implementation.

Multimedia Appendix 7 Enrollment of new users per day between May and September 2018 and the cumulative number of users in the period. The vertical dashed lines indicate media coverage of The Tick App.

Multimedia Appendix 8 Age and gender distribution of users of The Tick App.

Multimedia Appendix 9 Results of the multiple correspondence analysis (MCA), including frequent outdoor activities (peridomestic and recreational), having an outdoor job and owning a pet.

Multimedia Appendix 10 The recurring users’ profile including demographic variables, frequent outdoor activities (occupational, recreational and peridomestic), and owning a pet as reported in the enrollment survey.

## Abbreviations

CDC: Centers for Disease Control and Prevention
EMA: ecological momentary assessment
GPS: global positioning system
IQR: interquartile range
IRB: Institutional Review Board
IRR: incidence rate ratio
MCA: multiple correspondence analysis
mHealth: mobile health
OR: odds ratio
