# Supplementary material for: Usability and Feasibility of a Smartphone App to Assess Human Behavioral Factors Associated with Tick Exposure (The Tick App): Quantitative and Qualitative Study
Source: JMIR Mhealth Uhealth. 2019 Oct 24;7(10):e14769. doi: 10.2196/14769 (PMC6913724; doi:10.2196/14769)
Supplement: Multimedia Appendix 1 [file mhealth_v7i10e14769_app1.pdf]

Multimedia Appendix 1. Phase 1 and 2: Pre-prototype and prototype design.

We tested "GeoQuestion" on Block Island, RI, an endemic area for Lyme disease, during the summer of 2017. This app aimed to collect human behavioral and self-reported tick exposure data. It consisted of a one-time survey that aimed to establish the baseline risk and two brief daily surveys about the user's outdoor activities and tick exposure: one that appeared randomly during the day (9 am to 5 pm) and one retrospective survey each evening for the duration of the summer. The original concept of the app was expanded in the prototype phase to include more interactive features for the users, including the ability to: report details about their tick encounters; submit pictures of ticks to help identify different species and life stages; and access information about prevention methods as well as about tick characteristics and tick ecology. We integrated the feedback provided by the users of the pre-prototype app (i.e., contextual inquiry) with the scientific objectives of The Tick App as a tool to collect behavioral data regarding tick encounters (identify research objectives). Then, in an effort to engage with the users, their values and motivations were considered when designing the content of the app (surveys, educational materials, etc.) (i.e., value specification).

Testing of the prototype: pre-operationalization feedback

Before The Tick App launch on May 28th 2018, we conducted focus groups on Staten Island, NY, Block Island, RI, and in Eau Claire, WI. The goal was to assess people's general knowledge of ticks and tick-borne diseases, as well as to gain insights into the likelihood of people using the app and the best approaches to promote it. We recruited participants through advertisements in local public places (i.e. flyers in public buildings).

The desirable functionalities mentioned during focus groups included tick identification services, risk assessment and presence of ticks in their neighborhood or town, and news or information on ticks and Lyme disease. Tick identification was implemented in the "Tick ID", "Report a Tick" and online picture submission; educational information was included in the "Tick Safety" feature. Risk information (i.e. acarological risk) for specific neighborhoods was not included due to the challenges of obtaining accurate

measures of risk at such a fine spatial scale, concerns that risk maps based solely on user reports of tick activity would be incomplete and often inaccurate, and sharing of personal identifiable information (longitude – latitude, address, zipcode and/ or county) would be a HIPPA violation. Regarding the "Tick Diary", some concerns were raised about compliance in completing daily surveys, but all participants recommended against daily reminders to complete the surveys. Reminders were adjusted following their recommendations (i.e, daily but until 15 daily surveys were completed). Social media, local newspapers and promotional material in public places were mentioned as the best recruitment strategies, as well as engaging and reaching out to groups that could be interested in the app. During the pilot testing of the prototype, users commented on the design, content and accessibility of The Tick App. Overall, users commented positively on the app design and mentioned it was appealing and accessible to users. Conversely, the general consensus was that the information was insufficient regarding the objective of the app and why it would be useful to users, and that it should be more clearly stated in the first section of app (before agreeing to the consent form). We adjusted the content of The Tick App based on the feedback, which also guided the content design of the "Help" section of the app.
