# Supplementary material for: Usability and Feasibility of a Smartphone App to Assess Human Behavioral Factors Associated with Tick Exposure (The Tick App): Quantitative and Qualitative Study
Source: JMIR Mhealth Uhealth. 2019 Oct 24;7(10):e14769. doi: 10.2196/14769 (PMC6913724; doi:10.2196/14769)
Supplement: Multimedia Appendix 4 [file mhealth_v7i10e14769_app4.pdf]

<u>Multimedia Appendix 4.</u> Technical appendix of the backend components of The Tick App.

The backend components consist of two main features that run on a server to facilitate data collection: the portal server and data storage features. The portal server receives the data from the smartphone application, while the data storage feature checks it against the participant manager (which provides user authentication). Then, the portal server stores the data in a database that can handle very large data sets (Data storage feature).

<u>Portal server</u>

The Tick App v1.0 was built using Java for Android and Swift 3.0 for iOS. The app retrieves and transmits data through SSL-encrypted API calls to Linux servers running Apache, PHP, and MySQL maintained by CHESS at the University of Wisconsin – Madison College of Engineering. To ensure that the Tick App still functioned without a network connection, content and survey content were stored on the device and removed if a participant logged out. Any surveys filled out when the device did not have a network connection were cached on the device until a connection became available.

Version 2.0 of The Tick App will be built using the Ionic framework version 3 allowing developers to build apps for Android and iOS as well as a website using a single codebase.

<u>Data storage and privacy</u>

Data collected through the app is stored in a secure, limited access server maintained at the University of Wisconsin - Madison College of Engineering. Participants were identified with a unique number automatically generated when the participant creates an account with the app. Profile information is stored in a separate file from the survey responses. The time and location stamp linked to the report-a-tick and daily questionnaire did not contain other personal or sensitive information.

The data was distributed to the study team by the CHESS study coordinator through secure data sharing platform (i.e., UW Box) upon request of team members. Data was only ever handled by HIPAA trained study staff listed on the IRB and data maintenance / destruction will follow guidelines as set in the IRB. The Tick App uses regulatory compliant software and systems to ensure privacy and data security. Participants were unable to access temporarily stored data on their devices and temporarily stored data

was deleted from users' devices after transmission to CHESS servers. Sessions containing PHI and PII were encrypted from front-end and back-end listeners for transport encryption. CHESS routinely evaluates certificates and session negotiation policies and keep them consistent with the PHI & HIPPA best practices. Any individually identifiable information was removed prior to analysis.

Survey design

All surveys consisted of one to seven questions per page (i.e., screen) depending on the length of the question. We used adaptive questionnaires (i.e., some questions were displayed conditionally based on response to other questions) in order to reduce the number of screens and increase completion of the survey. The number of screens varied from 13 in the enrollment survey, 4 in the "Tick Diary" and "Report a Tick". Except for the questions regarding gender, age and location, all other questions were not mandatory and no completeness checks were applied. All items provided a none-response option when relevant. Users were able to edit their answers using the back button before submitting the survey.
