# Supplementary material for: Usability and Feasibility of a Smartphone App to Assess Human Behavioral Factors Associated with Tick Exposure (The Tick App): Quantitative and Qualitative Study
Source: JMIR Mhealth Uhealth. 2019 Oct 24;7(10):e14769. doi: 10.2196/14769 (PMC6913724; doi:10.2196/14769)
Supplement: Multimedia Appendix 5 [file mhealth_v7i10e14769_app5.pdf]

University of Wisconsin-Madison & Columbia University Consent to Participate in Research

Invitation

We invite you to take part in a research study titled “The Tick App”. The study is a collaboration between Dr. Maria Diuk-Wasser from Columbia University and Dr. Susan Paskewitz from the University of Wisconsin – Madison. We are inviting you because you are a resident or visitor of a high-risk Lyme disease area (Northeast and Midwest of the United States).

The purpose of this consent form is to give you the information you need to decide whether you want to participate in the study. We invite you to read through the question and answers related to this study. If we address all your questions that you might have about this study, and you have no further concerns, then you can decide if you want to be in the study. This process is called “informed consent,” and by signing at the end, you agree to participate.

The "TickApp" is intended to be informational; it provides no clinical assessments or diagnoses. Please contact your doctor if you have any medical concerns.

Why are researchers doing this study?

The purpose of this study is to better understand exposure to ticks and tick-borne diseases. This research is being done because Lyme disease is the most common vector-borne disease in the United States. Our behavior can influence the chances of being bitten by a tick and the information provided will help us design integrated control strategies to prevent diseases transmitted by ticks. Funding for this study is provided by the Centers for Disease Control.

What will happen in this study?

If you decide to take part in this study, you will be asked to share your daily activities with us and other information related to tick exposure. You will receive an enrollment survey that will take less than 10 minutes to complete. You will then receive a weekly to monthly message to start your tick diary. The tick diary, or activity report, should take less than a minute to complete. It asks if you or a household member encountered a tick and what you did that day. During submission it records, date, time and weather. When you start the tick diary, you will receive a daily reminder until you complete 15 reports. During the days that you are receiving daily reminders, the app will be tracking your location by GPS as frequent as every 15 minutes and as precise as 10ft within your actual location. You can turn this off in your phone settings.

You will receive a post-season survey in the fall that will take less 5 minutes to complete. In the post-season survey, you will be asked about the past spring and summer, if you are willing to participate in the following season and how we can improve the app. Clicking “Yes” to participation in the next year gives us permission to retain your e-mail address so we can contact you. You may skip any question on the questionnaires that you do not wish to answer. Lastly, you may be approached to participate in a focus group on the app-experience and separate information will be provided for this.

Will being in this study help me in any way?

You may benefit from the app reminders and educational material, as well as from sharing your experience and perspective with researchers and peers who value your input. Your participation in the study may benefit other people in the future by helping us learn about the risk factors for tick borne disease and helping us design better methods that prevent tick bites and tick-borne disease.

What are the risks?

We believe there are no risks associated with this research study; however, as with any online

related activity, there is a risk that your information could become known to someone not involved in this study. To the best of our ability your answers in this study will remain confidential. We will minimize any risks by keeping data on HIPAA compliant servers and by de-identifying demographic characteristics from your survey responses. That means that any personal information that could identify you would be decoupled from your answers and both databases will be linked by a code; that code will only be available for the researchers involved in the study. Also, your location/geographic information could identify you, but we will ensure that this data is only accessible by researchers with an encrypted key. In the final dataset, we will intentionally obscure coordinates, with true coordinates maintained in a separate file.

Potential stressors (fear or anxiety) may appear as a result of participating in this study as you become more aware of the risk of tick-borne diseases. Information regarding tick-borne diseases and best practices to prevent tick exposure are included in the app to help you take control when feeling unsure in a particular situation.

Who can access my information?

Members of the UW-Madison and Columbia University research team. Also, Institutional Review Boards (IRBs) from the University of Wisconsin-Madison and Columbia University, and the federal Office of Human Protections may obtain access to data from this study. Data will be labeled in a way so that no one can identify which answers came from you. After the study, data will be labeled in a way so that no one can identify which answers came from you. This data will remain securely stored, it does mean that we cannot remove your answers at a later time as we cannot identify which file is yours.

Will being in this study cost me anything?

The app is free. You will have to pay for your cell phone data.

What if I don’t want to participate now or after enrolling?

Participation is voluntary and you may discontinue participation at any time without penalty or loss of benefits. If you already submitted information and you want this removed, you need to contact the research team.

What if I have questions?

If you have questions about this research, please contact the lead researcher for your region, Midwest region (IO, IL, MI, MN, WI) Dr. Susan Paskewitz (608) 262-1696 or smpaskew@wisc.edu, North East region and other states Dr. Maria Diuk-Wasser (212) 854-3355 or mad2256@columbia.edu. If you have any questions about your rights as a research subject or have complaints about the research study or study team, contact UW Health Patient Relations at 608-263-8009 or the Columbia University Human Research Protection Office at (212) 851-7040 or askirb@columbia.edu.

Agreement to participate in the research study

By clicking ‘I agree’ below you are indicating that you are at least 18 years old, have read and understood this consent form and agree to participate in this research study. Please print a copy of this page for your records. A copy of this page will be available for you to access in the app at any time.

I AGREE

I DO NOT AGREE

Will be placed on Tick App business card / simple small folding flyer

Who can participate?
Anybody older than 18 years old. If you live in an area with ticks, you are invited to participate.

What is the study about?
In one word, ticks. You are probably familiar with ticks and heard about the diseases they can transmit through a tick bite, like Lyme disease. We study how human behaviors and activities impact their exposure to ticks.

What is in the app? The Tick App will ask you about your activities, your environment and possible prevention strategies in the enrollment survey. The app contains a quick Tick ID guide and helpful information on ticks and the diseases they transmit. You can also report-a-tick and you will be asked to share your daily tick encounters (or lack thereof) and whereabouts.

When is the study occurring?
May - October 2018, 2019 and 2020

Why is the study important?
The results of this study will be used to develop more effective strategies, tailored to each area, to prevent tick bites and the diseases that humans can get from ticks. For this reason, it's key to understand where and how are people getting exposed to ticks.

How can I participate?
Visit our website www.thetickapp.org, you will find instruction on how to download the app for your smartphone there or you can enroll in the online version of the study.

What if I have further questions?
Questions about the study can be directed to Maria Diuk-Wasser, principal investigator: mad2256@columbia.edu.

Visit our website: www.thetickapp.org

THE TICK APP

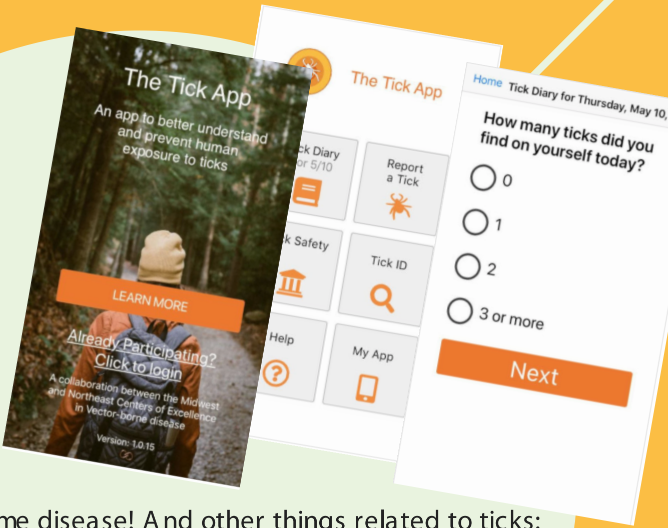

W hat is it about? Lyme disease! And other things related to ticks: how to identify them, how to report them and, most importantly for the research team, it asks about your whereabouts and tick encounters.

This is research? Yes, and it also reminds you to check for ticks and take precautions to not get bitten!

Download on the App Store

GET IT ON Google Play

What do I need to do? How can I participate?

1. Download the app (>18y old)
2. Complete the consent form and enrollment questions
3. Start filling in daily tick diaries

More information: www.thetickapp.org or tickapp@wisc.edu

What is in it for me? Citizen Science and more!

1. Your shared information will help us better understand where and how are people at risk of finding ticks.
2. You will have a reminder to check for ticks and information on how to prevent tick bites.
3. You can report a tick to us at any time and we'll identify it for you.

What's in The Tick App?

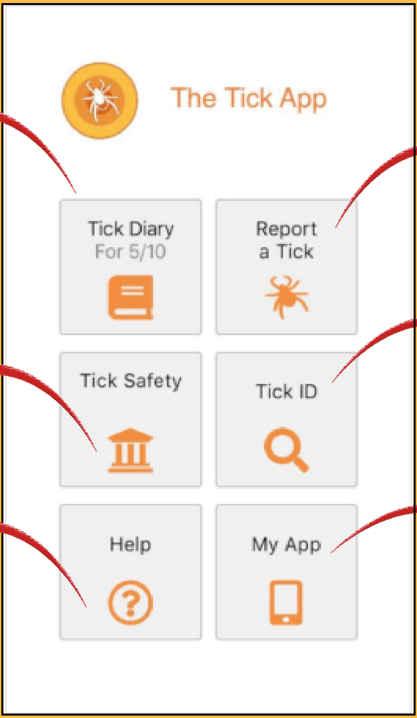

Complete the daily activity log for 15 days **here**. *Even if you don't find ticks!*

If you find a tick, you can report it **here**.

Learn how to prevent tick bites!

Learn how to identify ticks!

Learn how to use the app **here**

Keep track of your tick reports and tick diary entries **here.**

Why we want to know about your daily activities?

Knowing where the ticks are and how many it's not enough to understand where and how are people getting exposed to ticks.

We want to better understand how people's activity patterns affect the risk of tick exposure and design interventions that takes this into account.

By completing the tick diary for 15 days, even if you don't find ticks, you will help us better estimate how many people are at risk. This data is confidential and your personal information is decoupled from your activity log.
