# Supplementary material for: Usability and Feasibility of a Smartphone App to Assess Human Behavioral Factors Associated with Tick Exposure (The Tick App): Quantitative and Qualitative Study
Source: JMIR Mhealth Uhealth. 2019 Oct 24;7(10):e14769. doi: 10.2196/14769 (PMC6913724; doi:10.2196/14769)
Supplement: Multimedia Appendix 6 [file mhealth_v7i10e14769_app6.pdf]

Multimedia Appendix 6. Guiding questions for focus groups conducted prior and after The Tick App implementation.

<u>Before The Tick App launch – May 2018</u>

About the study:

- From your point of view, how do think our tick app study on behavioral data can contribute to reduce the human risk of tick-borne diseases.
- Do you have any doubts or questions about our study and its goals?
- How can we make the goals of the study clearer?
- Do you have suggestions about the house visits and work on the backyard of houses?
- What do you think will motivate people to participate?
- How is the best way to advertise our study and inform the community about it?

About the app: Use the app and capture general comments and ideas

- Do you use apps regularly on your phone?
- Did you find it easy to use or was there anything you found difficult when using the app?
- Which parts of the app did you find most confusing?
- Which parts of the app did you find easier to use?
- How do you feel about the length of the study and the frequency of the surveys?
- What features would make the app easier to use?
- Apart from the app, what else do you think we could do to gather and give information?
    a. E-mail surveys
    b. Web page with the surveys and information
    c. Text message services
    d. Paper surveys

<u>After The Tick App launch – September 2018</u>

The Tick App - Discovery

- Do you use apps regularly on your phone?
- When and how did you learn about the Tick App?
- Why did you decide to download the Tick App? Or why did you decide not to download the Tick App? (Did you participate on paper or online?)
- Was it easy to find?
- What did you think of the enrollment process? Were the goals of the study clear?

The Tick App - Use

- What did you look for first in the home screen? / What did you do when you entered the home screen?
- What did you think the researchers wanted you to do?
- How did you use the app?
- Which parts of the app did you find most confusing?
- Which parts of the app did you find easier to use?
- What features would make the app easier to use?

ALTERNATIVES:

- Tick diary:
    - What did you think Tick Diary meant?
    - How did you use the tick diary?
    - Did you complete any tick diaries?
    - How did you use it?
    - Do you think the goals of the tick diary were clear?

    - How can we make it clearer/incentivize people to use it?
- Tick report:
    - What did you think Tick report meant?
    - How did you use the tick report?
- Tick safety:
    - How did you use information?
    - Did the information there had an impact in your preventive practices?

The Tick App - Improvements (guiding questions)

- What did you think of the home screen?
- Were the different buttons in the app self-explanatory?
- What else would you like to see in the app?
- How would an app like this be most useful to you?
- Would you recommend the app to your family and friends?

Research incentives

- What do you think is the best way to promote the app?
- What would make continuous use of the app in the spring and summer more attractive?
- Would you like to receive reminder to check for ticks when walking in/out of parks or later in the day if you had been in a park?
- What would you think the best incentive to complete the tick diaries would be?
