# Supplementary figures and images for: Usability and Feasibility of a Smartphone App to Assess Human Behavioral Factors Associated with Tick Exposure (The Tick App): Quantitative and Qualitative Study
Source: JMIR Mhealth Uhealth. 2019 Oct 24;7(10):e14769. doi: 10.2196/14769 (PMC6913724; doi:10.2196/14769)

Number of new users

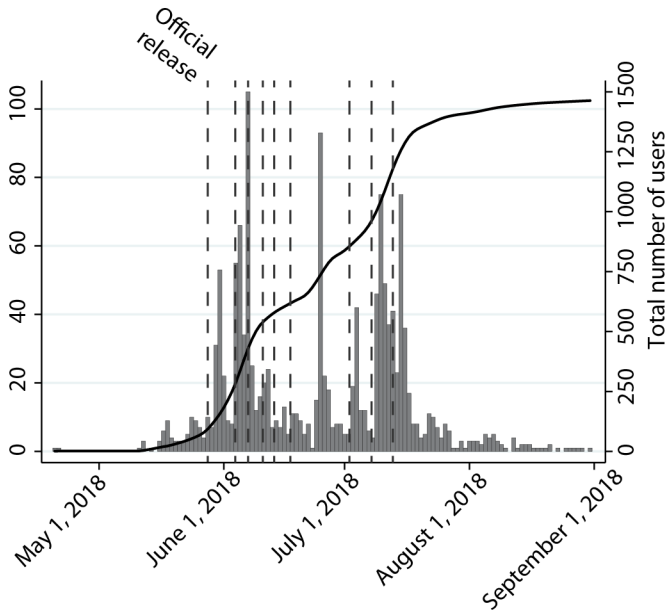

Supplement: Multimedia Appendix 7 [file mhealth_v7i10e14769_app7.pdf]

Proportion of users

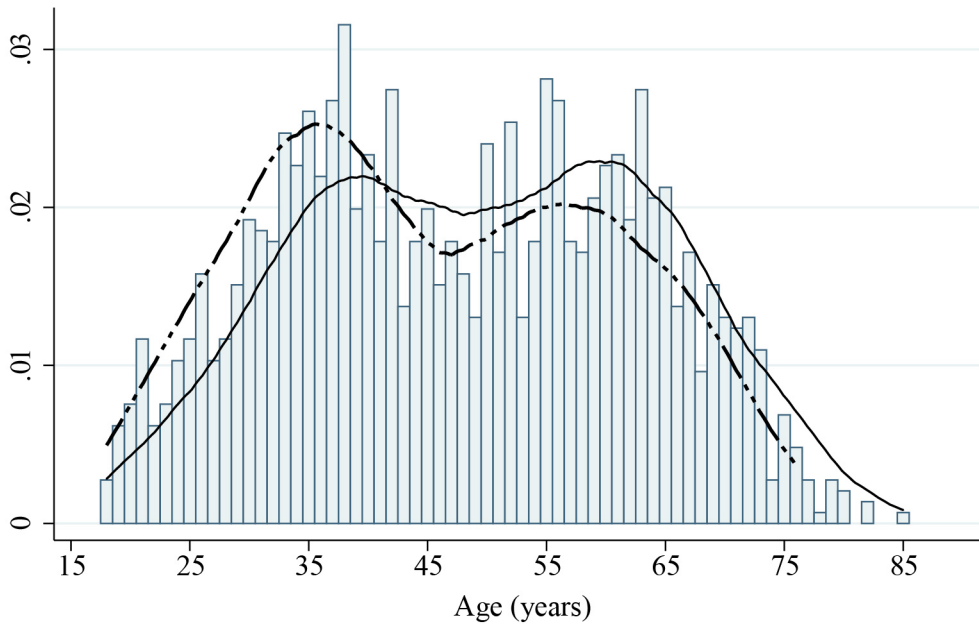

— Females    - - - Males

Supplement: Multimedia Appendix 8 [file mhealth_v7i10e14769_app8.pdf]
