# Supplementary material for: Usability and Feasibility of a Smartphone App to Assess Human Behavioral Factors Associated with Tick Exposure (The Tick App): Quantitative and Qualitative Study
Source: JMIR Mhealth Uhealth. 2019 Oct 24;7(10):e14769. doi: 10.2196/14769 (PMC6913724; doi:10.2196/14769)
Supplement: Multimedia Appendix 9 [file mhealth_v7i10e14769_app9.pdf]

<u>Multimedia Appendix 9.</u> Results of the multiple correspondence analysis (MCA), including frequent outdoor activities (peridomestic and recreational), having an outdoor job and owning a pet.

Dimension 1 (89.5% of the total inertia)		
Categories	Dimension 1 coordinates	Contribution to inertia
Owning a pet		
No	0.89	0.07
Yes	-0.45	0.03
Working or volunteering outdoors		
No	0.82	0.09
Yes	-1	0.12
Frequent outdoor recreational activity		
No	2.1	0.25
Yes	-0.7	0.08
Frequent peridomestic activity		
No	1.6	0.22
Yes	-0.9	0.12
