# Supplementary material for: Usability and Feasibility of a Smartphone App to Assess Human Behavioral Factors Associated with Tick Exposure (The Tick App): Quantitative and Qualitative Study
Source: JMIR Mhealth Uhealth. 2019 Oct 24;7(10):e14769. doi: 10.2196/14769 (PMC6913724; doi:10.2196/14769)
Supplement: Multimedia Appendix 10 [file mhealth_v7i10e14769_app10.pdf]

Multimedia Appendix 10. The recurring users' profile including demographic variables, frequent outdoor activities (occupational, recreational and peridomestic), and owning a pet as reported in the enrollment survey. The Odds ratios and *P*-values of the bivariate logistic regressions assessing the likelihood of being a recurring user vs. no interacting with The Tick App beyond the enrollment survey.

	Percentage	Total n	OR (CI_{95})	P
**Gender**		721		
Male	49.6		1.0	-
Female	49.5		1.0 (0.8-1.2)	0.8
Other/prefer not to say	0.9		0.4 (0.1-1.0)	0.04
**Age**		719		
18-24	6.5		1.0	-
25-34	16.3		0.8 (0.5-1.2)	0.3
35-44	20.2		0.8 (0.5-1.3)	0.4
45-54	19.9		1.1 (0.7-1.7)	0.7
55-64	24.5		1.2 (0.8-1.9)	0.4
65 or older	12.6		0.9 (0.5-1.5)	0.7
**Pet owner**		716		
No	32.5		1.0	-
Yes	67.5		1.1 (0.9-1.4)	0.2
Activities				
**Work or volunteer outdoors**		718		
No	52.1		1.0	-
Yes	47.9		1.3 (1.1-1.6)	<0.01*
**Frequent outdoor activities**		712		
No	19.5		1.0	-
Yes	80.5		1.7 (1.4-2.2)	<0.001**
**Frequent peridomestic activities**		715		
No	33.6		1.0	-
Yes	66.4		1.2 (0.9-1.5)	0.1

*0.01<p<0.05 **0.001<p<0.01
